# Markedly Elevated Serum IgE in a Patient With Thrombocytopenia and a TUBA8 Variant of Uncertain Significance: A Case Report

**DOI:** 10.1155/crii/4951640

**Published:** 2026-11-01

**Authors:** Seyed Hesamodin Nabavizadeh, Aida Askarisarvestani, Maryam Esmaeilbeig, Mahdi Shahriari

**Affiliations:** ^1^ Allergy Research Center Shiraz University of Medical Sciences Shiraz Iran sums.ac.ir; ^2^ Department of Allergy and Clinical Immunology Shiraz University of Medical Sciences Shiraz Iran sums.ac.ir; ^3^ Department of Pediatric Hematology and Oncology Shiraz University of Medical Sciences Shiraz Iran sums.ac.ir

**Keywords:** Immunoglobulin E, macrothrombocytopenia, TUBA8, whole exome sequencing

## Abstract

**Background:**

Inherited macrothrombocytopenia comprises a heterogeneous group of platelet disorders characterized by low platelet counts and abnormally enlarged platelets, with clinical presentations ranging from absent to mild bleeding tendencies. Multiple genes involved in platelet cytoskeletal architecture and thrombopoiesis have been identified; among them, TUBA8 was recently recognized to encode tubulin alpha‐8, a critical component contributing to microtubule polymerization and organization.

**Case presentation:**

The patient was a 12‐year‐old boy being treated for thrombocytopenia who presented with eczematous lesions and atopic symptoms. The patient had significantly elevated immunoglobulin E (IgE) of 9300 IU/mL. In whole exome sequencing; the patient revealed an autosomal dominant mutation in TUBA8 gene, a variant of unknown significance, which is known to cause macrothrombocytopenia.

**Conclusion:**

This report of the unusual coexistence of macrothrombocytopenia, markedly elevated serum IgE, and a heterozygous TUBA8 variant of uncertain significance (VUS) underscore the importance of evaluating extra‐hematologic features in tubulin‐associated disorders and warrant further functional studies to delineate the role of α8‐tubulin in immune homeostasis.

## 1. Introduction

Inherited macrothrombocytopenia represent a heterogeneous group of rare congenital platelet disorders characterized by variable degrees of thrombocytopenia and a platelet size spectrum ranging from slightly enlarged to giant. Clinically, these disorders range from asymptomatic incidental findings to mild mucocutaneous bleeding [1]. Although the thrombocytopenia and bleeding manifestations in these patients may be frequently mild, but associations with other clinically important situations such as hematologic malignancies in ANKRD26, ETV6, and RUNX1 variants or progressive renal failure and sensorineural hearing loss in MYH9‐related disease may exist [2].

Normal platelet biogenesis and function depend heavily on a proper cytoskeletal architecture and megakaryocyte differentiation. As a result, pathogenic gene variants can impair cytoskeletal regulation (ACTN1, FLNA, MYH9, and TUBB1), reorganization (GFI1B and NBEAL2), and surface glycoprotein expression in different stages of megakaryocyte differentiation (GP1BA, GP1BB, and GP9) [3–6]. Given the pivotal role of tubulin‐based microtubules in platelet cytoskeletal architecture, mutations in TUBB1 and TUBA4A and newly recognized TUBA8 genes have been linked with impaired platelet formation [7].

While TUBA8 has well‐established roles in brain development and spermatogenesis—with biallelic mutations implicated in neurodevelopmental disorders such as polymicrogyria—its heterozygous variants have only recently been linked to macrothrombocytopenia [8, 9].

To date, the phenotypic spectrum of TUBA8‐associated platelet disorders has remained strictly confined to hematological indices without documented evidence of immune dysregulation. Here, we present the case of a patient harboring a TUBA8 variant of uncertain significance (VUS) who presented with classic macrothrombocytopenia accompanied by markedly elevated serum immunoglobulin E (IgE) levels. This is an unusual coexistence in the classical phenotype of TUBA8‐associated disorders and highlights the potential immunological considerations in patients with tubulin‐associated inherited macrothrombocytopenias.

## 2. Case Presentation

We report the case of a 12‐year‐old boy. Written informed consent was obtained from the patient’s parents, who are the legal guardians. The patient is well‐developed and fully vaccinated who presented with recurrent episodes of epistaxis from 3 years prior to investigations. The patient had no purpuric or petechial lesions on skin examination and had no other source of bleeding. Also, he had a history of eczematous lesions being treated with topical corticosteroids and emollients by a dermatologist. No recurrent significant infections were noted in the patient’s past medical history. Performing diagnostic assessments, he had mild to moderate decreased platelet count ranging from 50 to 101 × 10^9^/L with a mean platelet volume (MPV) of 12.9 fL (reference range 7.0–11 fL). Repeated peripheral blood smears confirmed thrombocytopenia with the presence of giant platelets. Possible causes of thrombocytopenia were investigated including nutritional deficiencies, viral infections, such as HIV and hepatitis C and rheumatologic diseases, such as systemic lupus erythematous, which all were negative. The patient underwent bone marrow aspiration and biopsy, which revealed increased megakaryocytes. In the absence of a definitive diagnosis, the patient was empirically managed as a possible case of idiopathic thrombocytopenic purpura (ITP) or autoimmune thrombocytopenia and was treated with a trial of prednisolone 5 mg and tranexamic acid 500 mg every other day. However, the clinical response was not significant.

Concomitantly patient was suffering from chronic cough, rhinorrhea, and sneezing. Investigations revealed normal lung physical examination, normal chest X‐ray, and normal pulmonary function test. Skin prick tests for aeroallergen sensitization were done for assessing allergic rhinitis, which showed normal results. However, in allergic evaluations, the patient had an elevated IgE level of 9300 IU/mL. The patient received appropriate treatment for his allergic disease. To exclude parasitic infections as an endemic cause of elevated IgE in our region, stool parasitological examination was performed and yielded negative results. Nevertheless, the patient was treated empirically with anthelmintic drugs; however, IgE levels did not decline despite treatment with anthelmintics and adequate control of the presumed atopic disease.

Given the patient’s overall clinical presentation and mostly due to unexplained thrombocytopenia, whole exome sequencing was requested. Genetic analysis identified a novel heterozygous frameshift variant in TUBA8 ( ^∗^OMIM 605742), a gene associated with macrothrombocytopenia‐2 (MACTHC2; OMIM #619840). Whole‐exome sequencing revealed the variant NM_018943.3: c.749_750dupTG, which is predicted to result in a premature termination codon at the protein level, p. (Asp251TrpfsTer42). According to ACMG/AMP guidelines, the identified variant was classified as a VUS. To the best of our knowledge, based on a review of the available literature and the ClinVar database, this variant has not been previously described. Due to resource limitations, orthogonal validation of the variant by Sanger sequencing was not feasible.

## 3. Discussion

In this case report, we discussed a case who was investigated for unexplained thrombocytopenia. The patient underwent genetic analysis which revealed a novel mutation in the TUBA8 gene in the setting of isolated macrothrombocytopenia. These patients typically have thrombocytopenia with low platelet counts and abnormally large, irregularly shaped platelets and may be asymptomatic or present with minor bleeding symptoms [7, 10]. The index patient also exhibited markedly elevated serum IgE levels, which prompted more diagnostic workup to elucidate the underlying etiology.

This gene encodes Tubulin Alpha 8 protein that is important in the organization and polymerization of microtubules and cytoskeleton structure and retrograde Golgi‐to‐apparatus transport. This protein also plays an important role in cell proliferation and growth. Previous disorders associated with this condition were autosomal dominant isolated macrothrombocytopenia and polymicrogyria with optic nerve hypoplasia [9, 11, 12]. However, the association of this gene with elevated IgE has not been reported yet.

Elevated serum IgE is associated with allergic diseases, including asthma, allergic rhinitis, and atopic dermatitis; allergic bronchopulmonary aspergillosis; inflammatory disorders; parasitic infections; certain malignancies; and specific inborn errors of immunity. Although no universal IgE cutoff reliably distinguishes these conditions, atopic dermatitis is generally associated with the highest IgE concentrations among common allergic diseases [13].

In this patient, the markedly elevated total IgE level of 9300 IU/mL could be related to the underlying allergic condition. However, the degree of IgE elevation appears disproportionate to the severity of the patient’s allergic disease and is not adequately explained by the clinical manifestations alone.

Inborn errors of immunity characterized by the triad of eczematous lesions, thrombocytopenia, and elevated serum IgE levels such as primary hyper‐IgE syndromes (associated with STAT3, DOCK8, or PGM3 variants) and Wiskott–Aldrich syndrome (WAS) were key considerations in the differential diagnosis [14–17]. While the patient’s markedly elevated IgE levels initially raised concern for a hyper‐IgE syndrome, the lack of recurrent or opportunistic infections argued against such primary immunodeficiencies. Consistently, the patient had an NIH hyper‐IgE score of 14, and scores below 20 make classical STAT3‐hyper IgE syndrome unlikely [18].

Consequently, these findings suggest that the TUBA8 mutation may be associated with elevated serum IgE without manifesting as a classical inborn error of immunity.

Because the identified mutation is associated with an autosomal dominant inheritance, the presence of the disease in the parents might have been expected, which was not in this case. However, the absence of clinical manifestations does not exclude the possibility that the variant is present in either parent. A limitation of our study was the inability to perform Sanger sequencing to confirm the variant in the parents. Therefore, whether the variant is de novo or inherited cannot be determined in this case.

Pathogenic variants in TUBA8 have only recently been identified, and available clinical and current evidence regarding its broader clinical spectrum remains scarce. Uncovering new extra‐hematologic manifestations of TUBA8 variants may provide valuable insights into the fundamental role of microtubule dynamics in the immune system.

## 4. Limitations

The limitations of our clinical evaluation were the lack of confirmatory molecular and functional assays to validate the immunological consequences of the suspected genetic defect. We were unable to perform Sanger sequencing due to limited resources.

## 5. Conclusion

We report the unusual coexistence of thrombocytopenia, markedly elevated serum IgE, and a heterozygous TUBA8 VUS in a child without clinical evidence of classical hyper‐IgE syndrome. Although this observation may encourage further investigation of extra‐hematological findings in patients carrying TUBA8 variants, the available data do not establish a causal relationship between the variant and the elevated IgE level. Additional cases, segregation studies, and functional investigations are required to determine whether this coexistence is biologically meaningful or incidental.

## Funding

No funding was received for this study.

## Consent

Written informed consent for publication of the child’s clinical details was obtained from the patient’s parents, who are the legal guardians.

## Conflicts of Interest

The authors declare no conflicts of interest.

## Data Availability

The clinical data used to support the findings of this study are included within the article.
